# Assessment of foot care practice among adults with diabetes mellitus in Ogbomoso, Nigeria

**DOI:** 10.4314/ahs.v24i3.39

**Published:** 2024-09

**Authors:** Adeola Idowu, Isaac Amole, Adewumi Durodola, Olatayo Idowu, Stephen Adesina, Adepeju Adegoke, Akinsola Akinwumi

**Affiliations:** 1 Bowen University Teaching Hospital, Family Medicine; 2 Bowen University College of Health Sciences, Family Medicine; 3 Afe Babalola University College of Medicine and Health Sciences, Family Medicine

**Keywords:** Foot care practice, diabetic foot disease

## Abstract

**Background:**

The prevalence of diabetes mellitus (DM) has steadily increased as also the number of people bearing its complications. One of such complications is diabetic foot disease. Foot care is an integral part of diabetes self-care and preventive strategy for reduction of diabetic foot disease and ultimate amputation.

**Aim:**

To assess foot care practices among adults with diabetes mellitus accessing care at the Out-patient Clinic of the Bowen University Teaching Hospital, Ogbomoso, Nigeria.

**Methods:**

This was a cross-sectional study comprising of 384 adults aged 18 years and above with diabetes mellitus of at least 6 months duration. A systematic sampling technique was used to recruit the participants for this study. Nottingham Assessment of Functional Foot Care (NAFFC) was used to assess the foot care practices of the participants. Data analysis was done using Statistical Package for Social Sciences (SPSS) version 23.

**Results:**

Of the 384 participants recruited for the study, 321 (83.6%) had good foot care practice and 63 (16.4%) had poor foot care practice. Gender, level of education and medication use had significant association with foot care practice.

**Conclusion:**

Foot care practice was good in majority of the participants in this study. As such, clinicians need to be consistent in providing self-foot care practice education to prevent diabetic foot diseases.

## Introduction

Diabetes Mellitus is one of the non-communicable diseases and its prevalence has been increasing exponentially.^1^ There are diverse complications of DM and one of its long term complications is Diabetic foot disease (DFD). It is defined as a foot affected by ulceration that is associated with neuropathy and/or peripheral arterial disease of the lower limb in a patient with diabetes.^2^ The impact of DFD on patients is enormous, ranging from affectation of the physical wellbeing to socio-economic impact, psychological impact and reduction in overall quality of life.^3^,^4^

Around the world, one person undergoes amputation every 30 seconds due to DFD.^1^ Mortality rates after diabetic foot ulceration and amputation are high, with up to 70% of people dying within 5 years of having an amputation and around 50% dying within 5 years of developing a diabetic foot ulcer.^5^ The indirect costs that may be incurred from the management of DFD are prolonged hospital stay, overwhelming of hospital resources, loss of man power following the loss of a limb to foot gangrene, increase in morbidity and mortality and financial crisis in the family if the victim is the bread winner.^6^,^7^

Up to 85 % of amputations secondary to DFD can be prevented with adequate measures.^8^ One of such preventive measures is education of patients on self-foot care practices and exhibition of such by the patients in addition to good glycaemic control. Current guidelines by the American Diabetes Association (ADA) for patients with DM recommend annual screening of their feet and those identified as high risk should receive enhanced and focused foot care education towards good foot care practices.^9^ It has been shown that good foot care practices can reduce DFD by 50 to 60%.^10^ Therefore, the Diabetes Association of Nigeria (DAN) and ADA have recommended the following foot care activities; daily washing of the feet with soap, drying the feet with soft cloth, careful inspection of the feet for bruises or lacerations, not walking bare footed and careful selection of appropriate foot wears. 11

The aim of this study was to assess foot care practices among adults with diabetes mellitus accessing care at the Outpatient Clinic of the Bowen University Teaching Hospital, Ogbomoso, Nigeria. This assessment will assist clinicians to design possible interventional strategies towards the prevention of foot ulcers and ultimate amputation.

## Materials and methods

This study was a cross-sectional study conducted in the Outpatient Clinic of Bowen University Teaching Hospital, Ogbomoso, Southwest, Nigeria. Data were collected between March and June, 2018. Adult DM Outpatient Clinic runs every Wednesday (excluding public holidays) between 9.00am and 3:00pm. The average clinic attendance per week is 70. Activities engaged by the family medicine residents and nurses before patients' consultation included; diabetic health education and vital signs check. The study participants were 384 consenting adults aged 18 years and above with diabetes mellitus, who have been on treatment for at least 6 months. Pregnant patients, cognitively impaired patients and patients with active foot ulcers were excluded from the study.

The sample size of 384 for this study was calculated using the statistical formula;^12^


$$N=\frac{Z^{2}pq}{d^{2}}$$

N = sample size, p = prevalence of good foot care taken as 50.6%, (1) q = (1 − p), Z = standard normal deviation usually set at 1.96 which correspond to 95% confidence interval. d = degree of accuracy desired usually set at 0.05. Systematic sampling technique was used for selection of the participants. At least 24 participants were recruited per clinic appointment and sampling interval of 3 was used. The recruited patients' folders were tagged after contact and the tag was left in place throughout the study period to avoid multiple recruitments.

The questionnaire administered for data collection was divided into two (2) sections. Section A: socio-demographic characteristics and medical history of the participants which included; age, sex, marital status, religion, level of education, occupation, monthly household income, ethnicity, residential location, DM duration, medication in use, and other comorbid conditions. Section B: Nottingham Assessment of Functional Foot Care (NAFFC) questionnaire (Appendix A). The NAFFC questionnaire is a validated tool for assessment of the foot care practice in DM patients.^13^ It consists of 29 questions. Participants were asked to indicate frequency of occurrence of behaviour and rate them on a Likert scale ranging from 0-3. The maximum score obtainable in the Nottingham Assessment of Functional Foot questionnaire is 87. A total practice score of ≥ 50% (≥ 43.5/87) of maximum score implies good foot care practice while a score < 50% implies poor foot care practice.^14^

Data computation and analyses were done using Statistical Package for Social Sciences (SPSS) version 23 (IBM Corporation, Armonk, New York). Descriptive statistics, Chi-square test, and Fisher's exact were used for data analysis. Statistical significance was set at P ≤ 0.05. Ethical approval was obtained before the commencement of the study after the protocol was reviewed by the ethical committee of Bowen University Teaching Hospital, Ogbomoso, Nigeria.

## Results

The mean age of the study population was 59.3 ± 11.6. The age range with the highest proportion of participants was those who were above 60 years with 42.2%. A larger proportion of the participants 61.7% were females while only 38.3% were males giving a male to female ratio of 0.6:1. Majority of the participants (90.9%) were married. About 30.5% of the respondents had no formal education while 21.1% had tertiary education. Majority of the participants (68.2%) were urban dwellers. About 51.5% had coexisting medical conditions. The medical conditions identified included; hypertension which ranked the highest with a prevalence of 42.2%, osteoarthritis (5.7%), asthma (1.0%) and visual impairment (1.4%). Majority of the participants (65.9%) had duration of diabetes less than 5 years. The socio-demographic and medical details are shown in Table 1.

**Table 1:** Socio-demographic and medical characteristics of the participants (N = 384)

	Frequency	Percentage (%)
Age (years): ≤ 30	5	1.3
31-40	19	5.0
41-50	80	20.8
51-60	118	30.7
Above 60	162	42.2
Mean age = 59.3	Standard Deviation =	11.6
Gender: Female	237	61.7
Male	147	38.3
Marital status: Single	4	1.0
Married	349	90.9
Separated	6	1.6
Widow	25	6.5
Religion: Christianity	292	76.0
Islam	92	24.0
Education: No formal	117	30.5
Primary	114	29.7
Secondary	72	18.7
Tertiary	81	21.1
Ethnicity: Yoruba	367	95.6
Hausa	4	1.0
Igbo	13	3.4
Residence: Rural	122	31.8
Urban	262	68.2
Current Medications: Oral	230	59.9
Insulin	14	3.6
Both	140	36.5
Medical Condition: Absent	191	49.7
Hypertension	162	42.2
Osteoarthritis	22	5.7
Visual Defect	5	1.4
Asthma	4	1.0
Duration of Diabetes		
≤ 5 years	253	65.9
6 – 10 years	67	17.4
Above 10 years	64	16.7

Regarding the overall foot care practice of the study participants, 83.6% had good foot care practice and 16.4% had poor foot care practice. The NAFFC scores of participants ranged between 28/87 and 85/87 and the mean score was 51.2 ± 7.2. The most frequently practiced good foot care activity was not using a hot water bottle in bed (85.4%). This study revealed that 39.6% of the participants examined their feet daily and only 27.4% examined their feet more than once a day. (Table 2)

**Table 2:** Foot care practice

	Frequency	Percentage
Good foot care	321	83.6
Poor foot care	63	16.4
Total	384	100.0

A good number of the participants (58.6%) washed their feet more than once a day. About 49.0% of the participants cut their toenails about once a week while only 4.9% never cut their toenails. A high proportion of the participants, 31.8% often checked their shoes before they put them on. Only 16.1% of the participants often put a dry dressing on a blister when they got one while 58.1% never put a dry dressing on a blister when they got one.

Among the participants, 65.1% never walked outside in bare feet, and 37.2% never walked around the house in bare feet while 21.6% of the participants often walked around the house bare-footed. Majority of the participants (67.7%) wore slippers most of the time (Table 3). The study results showed that female gender, participants with primary education, and patients on oral medications had good foot care practice (P < 0.05) (Table 4).

**Table 3:** Isolated foot care practices

S/N	Questions	Answers	Points	Frequency	Percentage (%)
1	Do you examine your feet?	More than once a day	3	105	27.4
		Once a day	2	152	39.6
		Most days a week	1	22	5.7
		A few days a week	0	105	27.3
2	Do you wash your feet?	More than once a day	3	225	58.6
		Once a day	2	145	37.8
		Most days a week	1	11	2.8
		A few days a week	0	3	0.8
3	Are your toenails cut?	About once a week	3	188	49.0
		About once a month	2	165	43.0
		Less than once a month	1	12	3.1
		Never	0	19	4.9
4	Do you check your shoes before you put them on?	Often	3	122	31.8
		Sometimes	2	101	26.3
		Rarely	1	42	10.9
		Never	0	119	31.0
5	Do you check your shoes when you take them off?	Often	3	100	26.0
		Sometimes	2	81	21.1
		Rarely	1	62	16.2
		Never	0	141	36.7
6	Do you put a dry dressing on a blister when you get one?	Often	3	62	16.1
		Sometimes	2	82	21.4
		Rarely	1	17	4.4
		Never	0	223	58.1
7	Do you walk outside in bare feet?	Never	3	250	65.1
		Rarely	2	37	9.6
		Sometimes	1	72	18.8
		Often	0	25	6.5
8	Do you walk around the house in bare feet?	Never	3	143	37.2
		Rarely	2	41	10.7
		Sometimes	1	117	30.5
		Often	0	83	21.6
9	Do you wear sandals?	Never	3	230	59.9
		Rarely	2	25	6.5
		Sometimes	1	68	17.7
		Most of the time	0	61	15.9
10	Do you wear slippers?	Never	3	12	3.1
		Rarely	2	25	6.5
		Sometimes	1	87	22.7
		Most of the time	0	260	67.7
11	Do you wear pointed-toed shoes?	Never	3	258	67.2
		Rarely	2	21	5.5
		Sometimes	1	66	17.2
		Most of the time	0	39	10.1
12	Do you use a hot water bottle in bed?	Never	3	328	85.4
		Rarely	2	40	10.4
		Sometimes	1	7	1.8
		Often	0	9	2.4

**Table 4:** Association of foot care practice with socio-demographic and medical factors

	Good foot care N (%)	Poor foot care N (%)	χ^2^	P-value
**Age Group (years)**			**7.318**	**0.101**
≤ 30	5 (100.0)	0 (0.0)		
31-40	12 (63.2)	7 (36.8)		
41-50	67 (83.8)	13 (16.2)		
51-60	96 (81.4)	22 (18.6)		
Above 60	141 (87.0)	21 (13.0)		
**Gender**			4.994	0.025*
Male	115 (78.2)	32 (21.8)		
Female	206 (86.9)	31 (13.1)		
**Religion**			1.123	0.570
Christianity	241 (82.5)	51 (17.5)		
Islam	80 (87.0)	12 (13.0)		
**Level of Education**			**26.970**	0.01*
No formal	95 (81.2)	22 (18.8)		
Primary	110 (96.5)	4 (3.5)		
Secondary	58 (80.6)	14 (19.4)		
Tertiary	58 (71.6)	23 (28.4)		
**Medication Use**			17.856	0.001*
Oral	194 (84.3)	36 (15.7)		
Insulin	6 (42.9)	8 (57.1)		
Both	121 (86.4)	19 (13.6)		
**Diabetes Period**			0.542	0.762
≤ 5 years	209 (82.6)	44 (17.4)		
6 – 10 years	57 (85.1)	10 (14.9)		
Above 10 years	55 (85.9)	9 (14.1)		

(*) Statistically Significant

Bold values – Fisher's exact test

## Discussion

The highest proportion of the 384 participants in this study belonged to the age range of above 60 years and the age of the participants ranged from 30 to 85 years. The age distribution of the respondents in this study illustrated that majority of the participants with DM are usually in the older age group since the risk of Type 2 DM increases with age. This finding was in consonance with other related studies.^15^,^16^ Majority of the respondents were females with a prevalence of 61.7% with male to female ratio of 0.6:1. The larger proportion of females in the study could be due to the positive attitude of females to seeking health care.^17^ Consistent with this finding is the report of previous study in Senegal which reported a male to female ratio of 0.67:1.^18^ In this study, majority of the respondents had duration of DM of less than 5 years. The shorter duration of DM seen among the respondents may be due to late presentation to the hospital for early diagnosis and treatment as well as recall bias.

The foot self-care advice given to patients with diabetes mellitus is to reduce the likelihood of ulcer development which may progress to limb amputation.^19^ The overall foot care practice among majority of the participants was found to be good with a prevalence of 83.6%. This finding may be attributed to the commitment of the health practitioners on foot care education. In consonance with this report, Seid et al^14^ reported a good foot care prevalence of 54.6% in Northwest Ethiopia while Noaman et al^16^ reported 62.5% prevalence of good foot care practice in Baghdad. Conversely, Desalu et al^1^ also conducted multi-centre cross-sectional study in three tertiary hospitals in Nigeria, they reported that only 10.2% of the participants in their study had good foot care. This wide variation may be due to methodological differences as the tool that was used to assess foot care in the study was a self-developed tool unlike the NAFFC used in this study which is a validated tool for foot care assessment.

Assessment of the isolated foot care practice in this study revealed that majority of the participants washed their feet more than once per day. Desalu et al1 similarly reported that majority of the participants in their study washed their feet regularly. In this study, 49.0% of the participants cut their toenails about once a week. However, NAFFC failed to assess the type of tool that the participants cut their nails with. This is important because DM patients with retinopathy may injure themselves while using blade to cut their nails. The female participants were found to have higher proportion (86.9%) of good foot care practice (P < 0.05). This finding is not unexpected because females are known to keep to hospital instructions better than males.^17^ It was surprising to discover that participants with primary level of education had the highest proportion of good foot care practices. One would have expected that well educated participants would have highest proportion of good foot care practices as reported in the study of Desalu et al.^1^ This may be due to the fact that people with primary level of education are less involved in white-collar jobs^20^ making them have more time to visit the hospital, listen to foot care education, and practice what was learnt.

## Strength of the Study

Adequate sample size was recruited for the study. The instrument for foot care practice assessment (Nottingham Assessment of Functional Foot Care) was a validated tool for this purpose and not commonly used by other researchers in the region of study. Also, the questionnaire was interviewer-administered with pictorial representations such that information gotten was correct and complete.

## Limitation of the Study

1. The study was not a multicentre study such that larger proportion of participants with cultural diversity could be studied.
2. Considering that this is a cross sectional study, causality cannot be determined; also some of the data regarding the medical characteristics e.g. duration of DM and foot care practice may be subject to recall bias.

## Recommendation

There should be consistency in foot care education and periodic assessment of foot care practices among DM patients by clinicians.

## Conclusion

Foot care practice was good in majority of the participants in this study. As such, clinicians need to be consistent in providing self-foot care practice education to prevent diabetic foot diseases.
